# Post-Chemoradiation Lymphopenia and Baseline Eosinophil Counts as Prognostic Markers in Glioblastoma

**DOI:** 10.21203/rs.3.rs-7715242/v1

**Published:** 2025-10-12

**Authors:** Eric Giannaris, Geoffrey Sedor, Catherine S. Spina, Kristin Hsieh, Carl Elliston, Simon K. Cheng, Fabio M. Iwamoto, Guy M. McKhann, Michael B. Sisti, Jeffrey N. Bruce, Tony J. C. Wang, Matthew Gallitto

**Affiliations:** CUNY School of Medicine; Columbia University Medical Center; Columbia University Medical Center; New York Proton Center; Columbia University Medical Center; Columbia University Medical Center; Columbia University Medical Center; Columbia University Medical Center; Columbia University Medical Center; Columbia University Medical Center; Columbia University Medical Center; Columbia University Medical Center

**Keywords:** glioblastoma, lymphopenia, eosinophils, prognosis, hematologic markers

## Abstract

**Purpose:**

To evaluate whether post-treatment lymphopenia and white-cell differentials predict overall survival (OS) in glioblastoma (GBM).

**Methods:**

We retrospectively analyzed 93 GBM patients treated with standard surgery, radiotherapy (60 Gy in 30 fractions), and temozolomide. Clinical data (age, Karnofsky performance status, extent of resection, MGMT methylation, corticosteroid use, dose metrics) and hematologic indices (absolute lymphocyte count (ALC), neutrophil-to-lymphocyte ratio (NLR), absolute eosinophil (AEC) and basophil counts, etc.) were collected at baseline and up to 3 months post-chemoradiation.

**Results:**

Median OS in our cohort was 24.7 months (95% CI, 20.8–35.0). On univariate analysis, ALC < 0.75 ×10^3^/μL at 3 months was associated with shorter OS (HR 1.88; *p* = 0.042), and higher AEC correlated with improved OS at baseline (HR 0.71; *p* = 0.022) and at 1–2 months (HR 0.44 and 0.57; *p* = 0.046 and 0.016). In the combined multivariable model—controlling for age, extent of resection, MGMT status, steroid usage and dose metrics—baseline AEC remained independently prognostic (aHR 0.57; *p* = 0.016), whereas the association for 3-month ALC < 0.75 ×10^3^/μL attenuated (aHR 1.27; *p* = 0.49).

**Conclusions:**

Baseline eosinophils were independently associated with improved survival, while post-treatment lymphopenia was adverse only on univariate analysis. Such findings highlight the importance of host immunity and baseline eosinophils as a potential prognostic marker of OS in GBM and warrant larger, prospective studies on lymphocyte-sparing treatment strategies and prognostic marker validation.

## Introduction

Glioblastoma (GBM) is the most common malignant primary brain tumor in adults. Despite extensive trimodal therapy, including surgical resection, radiation, and chemotherapy, median overall survival is only 15 months, with a 5-year survival rate of 5% [1, 2]. Prognosis is often well-established by clinical and molecular factors such as age, KPS status, MGMT methylation, IDH mutations, and level of resection [2–6]. Yet survival can still vary widely among GBM patients with similar clinicopathologic features.

GBM patients are frequently immunosuppressed, reflecting both tumor-mediated immune evasion and therapy-related effects [7]. Lymphopenia is a common complication during and after chemoradiotherapy as circulating lymphocytes are extremely radiosensitive and undergo apoptosis within hours of systemic irradiation [8]. While many hematologic factors recover, lymphocyte depletion can persist for months [9]. Lymphopenia has been associated with inferior survival outcomes in several studies, suggesting that patients’ immune function during and after treatment could potentially impact long-term survival [10–12]. Similarly, the neutrophil-to-lymphocyte ratio (NLR) and lymphocyte-to-monocyte ratio (LMR) have been associated with worse outcomes in gliomas and GBM [13–16].

Beyond these markers, interest has recently expanded to other immune subsets, including eosinophils and basophils. *In vitro*, eosinophils have been associated with attraction towards glioblastoma cells and promotion of Fas/FasL–mediated apoptosis [17]. Clinically, in an analysis of GBM patients treated with bevacizumab, an increase in eosinophils during treatment correlated with improved survival [18]. Similarly, in a retrospective cohort of GBM patients, higher absolute eosinophil counts, and elevated lymphocyte-to-basophil ratios independently predicted longer overall survival [19].

In this study, we perform a retrospective analysis of hematologic parameters in a cohort of GBM patients to identify predictors of survival. In clarifying the prognostic impact of post-treatment lymphopenia and different white cell counts (including NLR, eosinophils, and basophils), therapeutic strategies can be targeted to preserve and improve immune function during standard treatment.

## Methods and Materials

### Patients

This single-institution retrospective analysis included 93 adult patients with pathology-proven glioblastoma (GBM) enrolled on the IRB-approved database clinical trial AAAM-2358 between 12/20/2011 and 06/20/2016. Patient inclusion criteria included GBM patients treated at our institution with maximally safe surgical resection followed standard radiation therapy for a total dose of 60 Gy delivered in 30 daily fractions delivered Monday - Friday with concurrent (75mg/m^2^/day) and adjuvant temozolomide (150–200 mg/m^2^/day, 5 days per 28-day cycle). Exclusion criteria included infection treated with antibiotics, prior radiation therapy to the central nervous system (CNS), incomplete course of radiation therapy, and a lack of recorded follow-up in the initial 3 months post-chemoradiation therapy.

Patient demographics and treatment details, including use of chemotherapy and steroids, were obtained from the electronic medical record. Hematologic assessment was conducted using the electronic medical record, collecting the complete blood count (CBC) with differential within 2 weeks of starting chemoradiation therapy (pre-CRT), within 2 weeks of completion of chemoradiation therapy (post-CRT) and monthly for the first three months after treatment. Dosimetric analyses were undertaken using Eclipse^®^ or Pinnacle software and patient pre-treatment CT scan generated at time of simulation. The volumetric analyses included evaluation of the following structures: planning target volume (PTV), brain parenchyma and calvarium. All data was stored according to IRB-approved best practices.

### Statistics

All statistical analyses were conducted in R (v4.3.1). Univariate analyses were conducted using Cox proportional hazards regression, computing hazard ratios with 95% confidence intervals and p-values with statistical significance defined as p < .05. Variables found to be significantly correlated on univariate analysis were further assessed in a multivariate Cox model to determine independent prognostic factors. We included one timepoint per biomarker and did not co-model composite ratios with their component cell counts. Covariates that were significant on univariate analysis or deemed clinically important in established literature were included in the multivariable model: extent of resection, MGMT methylation status, age at diagnosis, radiation treatment duration, and percentage of the brain receiving at least 60 Gy. The Kaplan-Meier analysis was used for survival analyses, using the log-rank test for significance.

## Results

### Patients

This cohort consisted of 93 GBM patients, 63% male, and 37% female with a median age of 59 years and median Karnofsky Performance Status 90 (Table 1). All patients received standard concurrent chemoradiotherapy, and the median radiotherapy duration or ‘package time’ was 42 days. All patients underwent maximally safe surgical resection, with gross total resection in 35%, subtotal resection in 57%, and biopsy in 6%. MGMT promoter methylation was present in 28% of patients, absent in 58%, and unknown in 14%. The median total duration of steroid use was 49 days, with 45% of patients requiring long-term steroids beyond 2 months into adjuvant therapy.

At the time of analysis, 73 patients had died (78.5%). The median overall survival was 24.7 months (95% CI 20.8–35.0 months). Due to the retrospective nature of the study, pre-treatment hematologic values were available for 90 (97%) patients, 91 (98%) patients immediately post-treatment, 75 (81%), 75 (81%) and 60 (65%) of the patients 1, 2 and 3 months after completion of chemoradiation therapy, respectively.

### Overall Survival

On univariate Cox analysis, several clinical factors and hematologic variables showed significant association with overall survival, summarized in Table 2. Extent of resection significantly correlated with improved survival outcomes as those who underwent subtotal resection (n = 54) and biopsy (n = 6) had worse survival *(HR 1.99, P = 0.0086; HR 4.17, P = 0.0046, respectively)* when compared to gross total resection (n = 33). MGMT methylation (n = 26) was also associated with improved survival *(HR 0.57, 95% CI 0.33–0.98, P = 0.042).* IDH mutation (n = 8) was also significantly associated with extended survival *(HR 0.34 if mutant vs. wildtype, P = .031)*, consistent with expectations despite low frequency.

Prolonged steroid use for greater than 60 days had significantly higher mortality than those who were able to discontinue steroid treatment *(steroid use < 60 days: HR .575, P = .0229).* Similarly, treating steroid duration as a continuous variable produced similar findings as the total number of steroid days was positively associated with worse overall survival *(HR 1.01 per day of use, P = .021).*

Radiation-related factors that correlated negatively with overall survival on univariate analysis included duration of radiation *(HR 1.12, P = 0.046)* and percent of brain tissue receiving at least 60 Gy *(HR 1.32, P = 0.026).*

**Figure 1:**
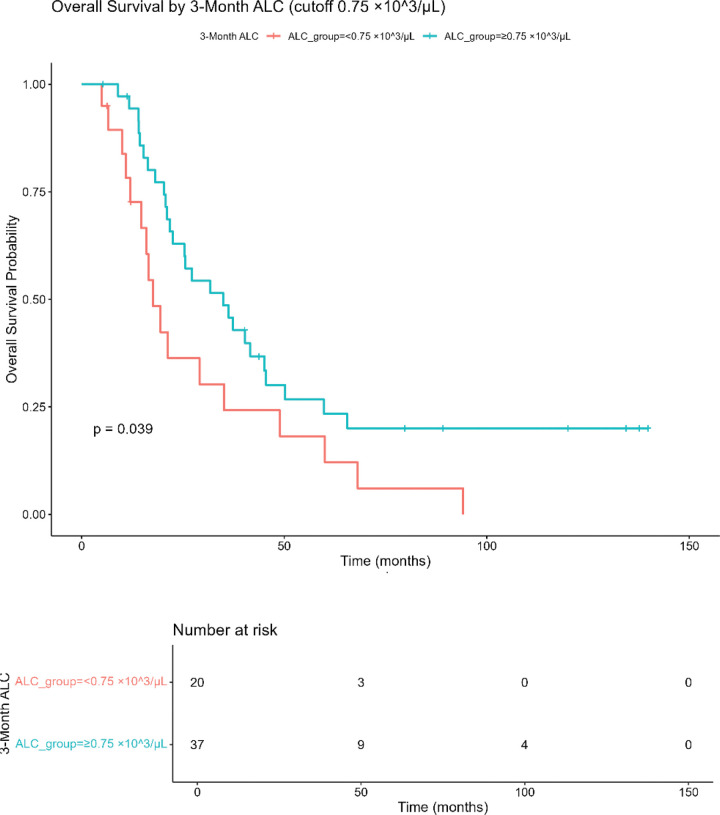
Kaplan-Meier overall survival by 3-month absolute lymphocyte (ALC) OS stratified by ALC < 0.75 vs ≥ 0.75 × 10^3^/μL measured 3 months after CRT. Numbers at risk shown below the plot; log-rank p displayed in panel. ALC = absolute lymphocyte count.

From a hematologic perspective, persistent post-treatment lymphopenia, defined in this study as ALC < 0.75 × 10^3^/uL at 3 months, had significantly shorter overall survival on univariate analysis *(HR 1.88, P = .042).* Figure 1 illustrates this difference as a Kaplan Meier survival analysis. The median overall survival for 3-month ALC ≥ 0.75 was *35.0 months (95% CI 22.5–50.2)* and for 3-month ALC < 0.75 × 10^3^/uL group was *17.6 months (95% CI 14.7–60.0).* The log-rank test was significant *(p = 0.039).*

**Figure 2:**
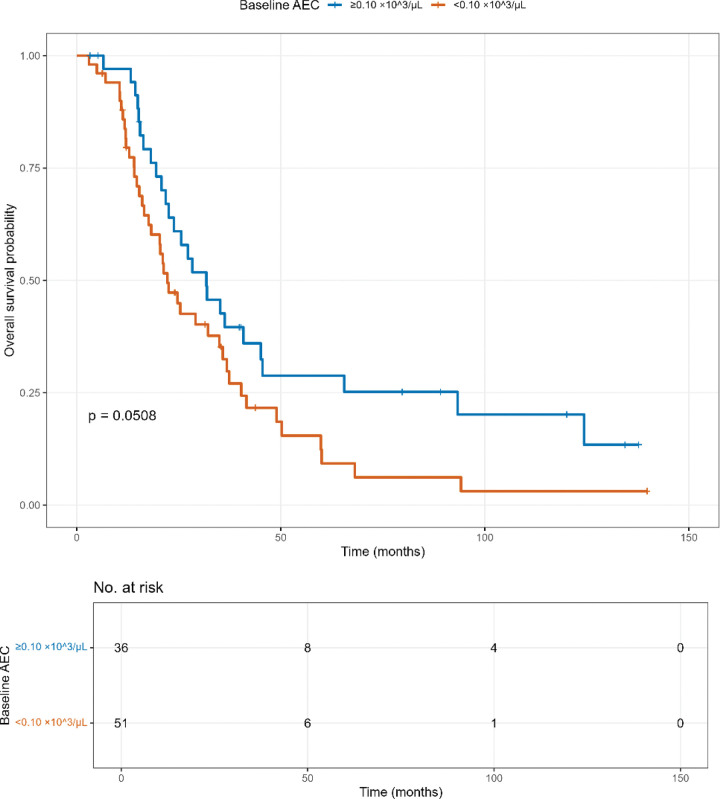
Kaplan Meier overall survival by baseline absolute eosinophil count (AEC) OS stratified by AEC < 0.10 vs ≥ 0.10 × 10^3^/μL at baseline (pre-CRT). Numbers at risk shown below the plot; log-rank p displayed in panel. AEC = absolute eosinophil count.

Moreover, peripheral eosinophil counts correlated positively with survival at baseline, 1 month, and 2 months on univariate analysis *(HR .71, P = 0.022; HR 0.44, P = 0.046; and HR 0.57, P = .016; respectively).* Patients who maintained higher levels of eosinophils before and after treatment were associated with improved survival outcomes. To further explore this finding, a Kaplan Meier survival analysis was conducted dichotomizing baseline absolute eosinophil count (AEC) at the mean for our cohort, 0.10 × 10^3^/uL (Fig. 2). The median overall survival for baseline AEC ≥ 0.10 × 10^3^/uL was *31.8 months (95% CI 22.5–65.6)* and for baseline AEC < 0.10 × 10^3^/uL was *22.2 months (95% CI 17.6–35.8).* The log rank test was borderline significant *(P = 0.0508).*

Upon multivariate analysis for 3-month ALC < 0.75 × 10^3^/uL and baseline eosinophils while adjusting for clinical confounders (Table 3), baseline eosinophils remained a significant independent prognostic marker *(HR 0.57, P = 0.016).* Subtotal resection/biopsy, MGMT methylation, and percent of brain tissue receiving at least 60 Gy were the only other variables which significantly affected survival in this model *(HR 3.59, P = 0.002; HR 0.43, P = 0.040; HR = 1.52, P = 0.050 respectively).* The effect of 3-month ALC < 0.75 × 10^3^/uL attenuated.

Other hematologic markers also showed prognostic value in univariate analysis. A higher neutrophil-to-lymphocyte ratio (NLR) measured 2 months after treatment was significantly associated with poorer overall survival *(HR 1.58, P = .0086)* on univariate analysis. To avoid collinearity with pre-specified predictor ALC, NLR was not included in the multivariable model.

We also evaluated basophils, monocytes, platelets, WBCs, and RBCs across baseline, 1-month, 2-month, and 3-month time points *(Table S1)* which did not show significant univariate associations with overall survival.

## Discussion

In this study, we comprehensively evaluated the prognostic significance of hematologic parameters in a cohort of GBM patients treated at a single institution. We found that baseline eosinophils were a significant predictor of survival and other immune cell subsets including lymphocytes may also correlate with outcomes. Our results confirm and extend prior observations on the detrimental impact of lymphopenia and highlight novel associations involving peripheral eosinophil count [10–12, 19].

### Lymphopenia and Immunosuppression

The negative effect of lymphopenia at 3 months post-treatment observed in our cohort is in accordance with accumulating evidence that profound post-treatment immunosuppression may facilitate tumor progression and worsens survival in GBM.

Clinically, strategies that attempt to avoid or mitigate treatment-related lymphopenia could potentially improve outcomes. Modern radiotherapy techniques that aim to spare circulating lymphocytes are being explored. Preliminary evidence suggests that reduced radiation target volumes and hypofractionation might result in decreased lymphopenia in glioblastoma [20, 21]. Similarly, reducing steroid exposure could preserve lymphocyte counts. Our data reinforces the notion that whenever clinically feasible, minimizing steroid exposure is beneficial as we found steroid use < 60 days was associated with significantly longer survival [5]. This may be due to steroids’ impact on lymphocyte and other hematologic counts. Therapies to improve lymphopenia may also be of interest for patients with GBM such as adoptive cell therapies or IL-7 treatments to boost T cell counts [22, 23].

### Eosinophils

One of the novel findings of our study is the protective association of higher eosinophil counts with patient survival. Only a few studies have examined eosinophils in GBM prognosis. Madhugiri et al. found that both absolute eosinophil count and the neutrophil-to eosinophil ratio were different between short-term and long-term survivors, with higher eosinophils in the latter [19]. They report eosinophils as an independent predictor of survival in multivariate analysis. Our results support this as baseline eosinophil count was independently prognostic, and eosinophil counts 1 and 2 months after treatment were strongly associated with overall survival on univariate analysis. Importantly, this association for baseline eosinophils remained after adjusting for clinical confounders and 3-month ALC.

Eosinophils are associated with allergic and parasitic infections via the Th2 pathway and IgE-mediated mechanisms. In cancer, tumor-associated eosinophilia has been observed in certain malignancies to improve outcomes. In GBM, it has been observed that patients with increased IgE and CD23 have better prognoses [24, 25]. There is also evidence that eosinophils can exert cytotoxic effects on tumor cells and facilitate the recruitment of T cells to tumor sites in other cancers. In GBM, Vieira et al. reported that in situ eosinophil infiltration in GBM specimens was associated with tumor cell apoptosis [17]. Moreover, corticosteroids are known to decrease eosinophils [26], which may further support the ill effects of steroid use on GBM patients. Further research to clarify the role of eosinophils in GBM prognosis is warranted and should continue to be an area of interest.

Limitations of our analysis include the retrospective nature of the study, including the reliance on manually extracting data from the electronic medical record. Additionally, patients were not excluded from our analysis with incomplete hematologic follow-up, thus our evaluation of hematologic parameters 3 months after included 65% (n = 60) of the total cohort. Another limitation is that we did not have longitudinal data on subsets of lymphocytes (CD4, CD8, etc.), which are established markers of immunosuppression. Lastly, our cohort includes eight IDH-mutant patients which were treated as GBM as per WHO guidelines at the time of treatment. These data should be viewed as hypothesis-generating as numerous markers and timepoints were explored. Confirmation of our findings warrants a larger, multicenter prospective study.

## Conclusion

In this retrospective cohort of GBM patients receiving standard trimodal therapy, lower baseline eosinophils were independently associated with shorter overall survival. Treatment-related lymphopenia was associated with overall survival on univariate analysis and Kaplan-Meier survival analysis but did not independently predict survival. These findings support the clinical relevance of peripheral eosinophil count and its role in host immunity when assessing outcomes in GBM patients. These results support judicious corticosteroid use, further evaluation of lymphocyte-sparing treatment strategies, and the significance of eosinophils as a potential prognostic marker in GBM.

## Supplementary Material

Supplementary Files

This is a list of supplementary files associated with this preprint. Click to download.

• PostChemoradiationLymphopeniaandBaselineEosinophilCountsasPrognosticMarkersinGlioblastomaSupplementals.docx

## Figures and Tables

**Table 1:** Patient Characteristics

Characteristic	N or Value
**Total patients**	93
**Gender – Male**	59 (62.8%)
**Gender – Female**	34 (36.2%)
**Age (years)**	59 (15.7)
**Karnofsky Performance Status**	90 (50–100)
**Extent of Resection**:	
Biopsy only	6 (6.4%)
Subtotal resection (STR)	54 (57.4%)
Gross total resection (GTR)	33 (35.1%)
**MGMT status**:	
Methylated	26 (27.7%)
Unmethylated	54 (57.4%)
Unknown	13 (14.0%)
**IDH status**:	
WT	82 (88.2%)
Mutant	8 (8.6%)
Unknown	3 (3.2%)
**Radiotherapy duration (days)**	42 (1)
**Total steroid use (days)**	49 (129)
**Deaths during study**	73 (78.5%)
**Median Overall Survival**	24.7 months (95% CI 20.8–35.0)

Data are n (%) unless otherwise indicated; continuous values are median (interquartile range (IQR)). KPS = Karnofsky Performance Status; EOR = extent of resection (GTR = gross total resection; STR = subtotal resection); MGMT = O-6-methylguanine-DNA methyltransferase; IDH = isocitrate dehydrogenase

**Table 2:** Univariate Cox regression analysis for overall survival: clinical and significant hematologic factors.

Variable	HR (95% CI)	P-value
Subtotal resection (STR) vs GTR	1.99 (1.19–3.32)	*0.0086*
Biopsy vs GTR	4.17 (1.55–11.22)	*0.0046*
MGMT methylated	0.57 (0.33–0.98)	*0.042*
MGMT unknown	3.63 (1.78–7.40)	*0.0004*
IDH mutation (yes vs no)	0.34 (0.13–0.90)	*0.031*
Steroid days (per day)	1.01 (1.00–1.01)	*0.021*
Steroid use < 60 days (yes vs no)	0.58 (0.36–0.93)	*0.023*
Brain RT V60	1.32 (1.03–1.69)	*0.026*
Age at diagnosis	1.012 (0.99–1.03)	0.16
KPS	0.99 (0.97–1.01)	0.36
RT duration ‘package time’	1.12 (1.00–1.24)	*0.046*
PTV 6000 (per 10 cm^3^)	1.02 (1.00–1.03)	*0.043*
ALC < 0.75 (3-mo post-CRT)	1.88 (1.02–3.46)	*0.042*
NLR (2-mo post-CRT) (per SD)	1.58 (1.12–2.23)	*0.0086*
AEC (baseline) (per SD)	0.71 (0.53–0.95)	*0.022*
AEC (1-mo post-CRT) (per SD)	0.44 (0.20–.99)	*0.046*
AEC (2-mo post-CRT) (per SD)	0.57 (0.36–0.90)	*0.016*

Entries are hazard ratios (HR) with 95% CI; HR > 1 indicates higher hazard (worse survival). ALC (3-mo) dichotomized at 0.75 × 10^3^/μL; AEC and other continuous hematologic indices are reported per standard deviation (SD) at the specified time point (baseline AEC SD = 0.106 × 10^3^/μL). p values from two-sided Wald tests. Time windows: baseline (pre-CRT), post-CRT (within 2 weeks), and 1–3 months post-CRT. ALC = absolute lymphocyte count; NLR = neutrophil-to-lymphocyte ratio; AEC = absolute eosinophil count; V60 = percent brain volume receiving ≥ 60 Gy; RT = radiotherapy; PTV = planning target volume.

**Table 3:** Multivariable Cox model for overall survival

Variable	Adjusted HR (95% CI)	p-value
AEC (baseline) per SD	0.57 (0.36–0.90)	*0.016*
ALC < 0.75 (3-mo)	1.27 (0.64–2.50)	0.493
Subtotal resection/Biopsy (vs GTR)	3.59 (1.60–8.08)	*0.002*
MGMT methylated	0.43 (0.20–0.96)	*0.040*
Brain V60 (per 10%)	1.52 (1.00–2.30)	*0.050*
Age (per year)	1.00 (0.98–1.03)	0.804
Steroid days (per day)	1.00 (0.99–1.01)	0.181
Radiation duration (days)	0.87 (0.69–1.10)	0.233

Multivariate model: ALC (3-mo) + AEC (per SD) + covariates. Adjusted for age, EOR (GTR [ref], STR/biopsy), MGMT (unmethylated/unknown [ref]. methylated), steroid days (continuous), RT duration (days), and brain V60 (per 10%). Entries are adjusted HR (95% CI); p values from two-sided Wald tests. Abbreviations as in Tables 1 & 2; baseline AEC SD = 0.106 × 10^3^/μL.

## Data Availability

The datasets generated and analyzed during the current study are not publicly available due to institutional and patient privacy restrictions. De-identified data may be available from the corresponding author on reasonable request and with approval.
